# Intravitreal Injectable Hydrogels for Sustained Drug Delivery in Glaucoma Treatment and Therapy

**DOI:** 10.3390/polym14122359

**Published:** 2022-06-10

**Authors:** Kassahun Alula Akulo, Terin Adali, Mthabisi Talent George Moyo, Tulin Bodamyali

**Affiliations:** 1 Department of Biomedical Engineering, Faculty of Engineering, Near East University, Mersin 10, Lefkoşa 99138, Turkey; kasalula2012@gmail.com (K.A.A.); mtgmoyo@gmail.com (M.T.G.M.); 2 Tissue Engineering and Biomaterials Research Center, Near East University, Mersin 10, Lefkoşa 99138, Turkey; 3 Nanotechnology Research Center, Sabanci University SUNUM, Istanbul 34956, Turkey; 4 Department of Pathology, Faculty of Medicine, Girne American University, Mersin 10, Girne 99428, Turkey; heytuls@gmail.com

**Keywords:** glaucoma, natural biopolymers, intravitreal injectable hydrogel, drug delivery systems

## Abstract

Glaucoma is extensively treated with topical eye drops containing drugs. However, the retention time of the loaded drugs and the in vivo bioavailability of the drugs are highly influenced before reaching the targeted area sufficiently, due to physiological and anatomical barriers of the eye, such as rapid nasolacrimal drainage. Poor intraocular penetration and frequent administration may also cause ocular cytotoxicity. A novel approach to overcome these drawbacks is the use of injectable hydrogels administered intravitreously for sustained drug delivery to the target site. These injectable hydrogels are used as nanocarriers to intimately interact with specific diseased ocular tissues to increase the therapeutic efficacy and drug bioavailability of the anti-glaucomic drugs. The human eye is very delicate, and is sensitive to contact with any foreign body material. However, natural biopolymers are non-reactive, biocompatible, biodegradable, and lack immunogenic and inflammatory responses to the host whenever they are incorporated in drug delivery systems. These favorable biomaterial properties have made them widely applicable in biomedical applications, with minimal adversity. This review highlights the importance of using natural biopolymer-based intravitreal hydrogel drug delivery systems for glaucoma treatment over conventional methods.

## 1. Introduction

Reports of the World Health Organization (WHO) indicate that nearly 80 million people have glaucoma globally, and around half of the people with the disease are unaware that they have it. This number is expected to rise to 112 million individuals by 2040 [1]. Glaucoma has become the second most example cause of irreparable visual impairment around the world. It is a multifactorial, imperceptible, and gradual neurodegenerative disease that affects the optic nerve [2].

In human physiology, the eye is a delicate and complex organ that is specialized for detecting and converting light stimuli into meaningful information that is structured into different sections—namely, the anterior and posterior sections [3]. The anterior chamber located in between the cornea and the iris is filled with aqueous humor made by the ciliary body [4]. Fluid from the anterior chamber flows out through the pupil and then reaches the eye’s drainage system, as well as the trabecular meshwork and a network of canals [5,6]. The vitreous chamber situated between the lens and the back of the eye contains a thick, gel-like fluid called vitreous humor or vitreous gel [7,8]. Proper drainage of this fluid helps the eye to keep internal pressure at a normal level, which is an active and continuous process that is needed for the health of the eye.

**Figure 1:**
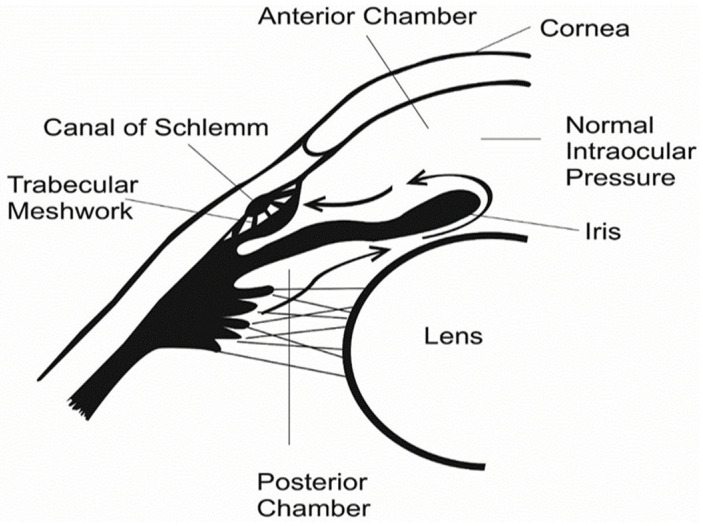
Physiology of a human eye with normal IOP.

In the human eye, the normal level of intraocular pressure (IOP)—as indicated in Figure 1—can be regulated by the balance between how much fluid is made and how much drains out of the eye in a given time [9]. In most types of glaucoma, the eye’s drainage system becomes clogged, so the intraocular fluid cannot drain [10].

The physiological optic cycle establishes the drainage flow through and out of the eye, and if any partial or total drainage obstruction occurs, elevation of the IOP may cause adverse effects on the retinal ganglion cells (RGCs) and RGC axons, causing glaucoma. The course of glaucomic progression is chronic and perdurable for a long time—usually asymptomatic, but gradually impairing the peripheral visual field before optimal damage [11].

The main causes of increased and abnormal elevation in IOP driving primary glaucoma remain undiscovered [12]; however, the causes of increased IOP leading to secondary glaucoma have been identified. Abnormally high IOP may be caused by advanced cataracts, inflammation, high or elevated blood pressure, optic tumors, diabetes, myopia, or hyperthyroidism [13]. Genetic and ethnic background, age, lifestyle, and narrowing retinal nerve fibers are risk factors that have been associated with glaucoma [14]. The disease has also been suspected to arise as a side effect of the prolonged use of corticosteroids [15].

Currently, there are various glaucoma treatments on the market that are designed to lower the IOP by lessening the production of aqueous humor or expanding non-trabecular fluid humor drainage with surgical- (i.e., implants and therapeutic surgeries) and pharmacological-based methodologies. Pharmaceutical therapeutics for the management of glaucoma through topical eye drops, ointments, and oral medications reduce elevated IOP; however, there are limitations, including low patient compliance and insufficient bioavailability [16]. To elevate bioavailability and improve patient compliance, advanced drug delivery mechanisms such as liposomes, microneedles, niosomes, dendrimers, ocular inserts, nanoparticles, and injectable hydrogels should be used [17].

To overcome the limitations of pharmaceutical therapeutics, several methods have been explored, including the use of in situ hydrogels, which potentially serve as delivery vehicles for nutrients, oxygen, and drugs to the targeted area [18].

Hydrogels play a significant role in upgrading the remedial adequacy of anti-glaucomic drugs, and are relevant in glaucoma treatment because of their reliable drug delivery applicability [19]. Polymers crosslinked with hydrophilic drugs can retain more than 90% water within the mesh of their porous network structure, thus aiding in the encapsulation of hydrophilic drugs [20]. Therefore, localized delivery of drug-loaded hydrogels can be achieved relatively more easily and less invasively than by implantation, and can reach the tissues that are difficult for conventional delivery methods to reach [21].

## 2. Glaucoma

**Figure 2:**
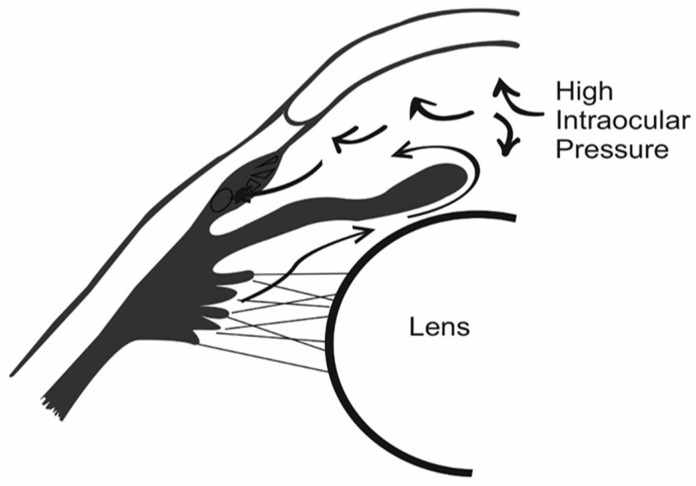
Open-angle glaucoma (chronic).

Glaucoma is classified primarily according to the severity and different causes of the ailment. The most predominant types are primary open-angle glaucoma (POAG) and primary acute angle-closure glaucoma (PACG) [22]. Gradual blockage of the eyes drainage channel—resulting in increased IOP, as shown in Figure 2—is the cause of POAG [23].

**Figure 3:**
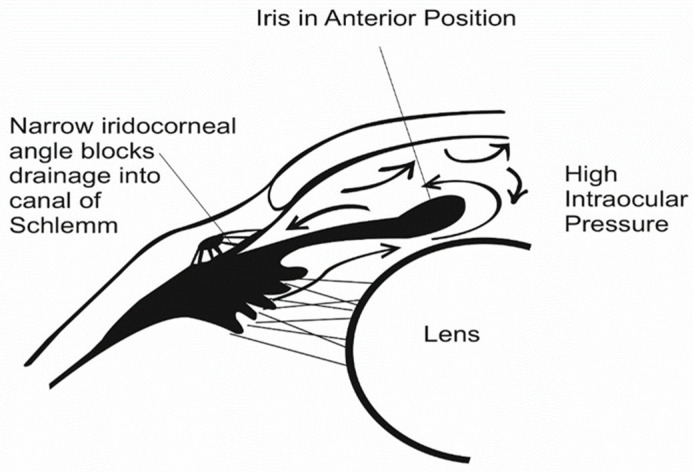
Blockage of Schlemm canal drainage.

Primarily, PACG is caused by elevation of the intraocular aqueous humor outflow at the closure angle, caused by mechanical occlusion of the iris tissue [24,25], or blockage of the Schlemm canal, as shown in Figure 3. Uveitis glaucoma, pigmentary glaucoma, and normal-tension glaucoma (NTG) are classified as secondary types of glaucoma [26].

## 3. Ocular Barriers

The eye’s anatomy and physiological structure comprise ocular barriers that are profound for defending its inner components from unfamiliar substances [27]. These ocular barriers include the blood–ocular barrier, tear film, conjunctiva, cornea, blood–aqueous barrier, and blood–retina barrier, which control the uptake of liquids. The anatomical barriers comprise conjunctiva, sclera, and the cornea, retina–blood–aqueous barrier, and blood–retina barrier. Secondly, all ocular mechanisms are protected by active physiological clearing systems. The systems are nasolacrimal drainage and pre-corneal tear secretion for the elimination of irritants, the blinking reflex, conjunctival blood flow, efflux transport, and choroid, which shield the eye from the effects of destructive drugs [28].

The principal objective of ophthalmological therapy is to bypass the defensive hindrances of the eye without affecting the surrounding tissue. These barriers forestall the transition, withholding, and bioavailability of some ophthalmologic medications by restricting ocular drug permeability to the foremost segments of the eye [29].

## 4. Current Therapies for Glaucoma

**Figure 4:**
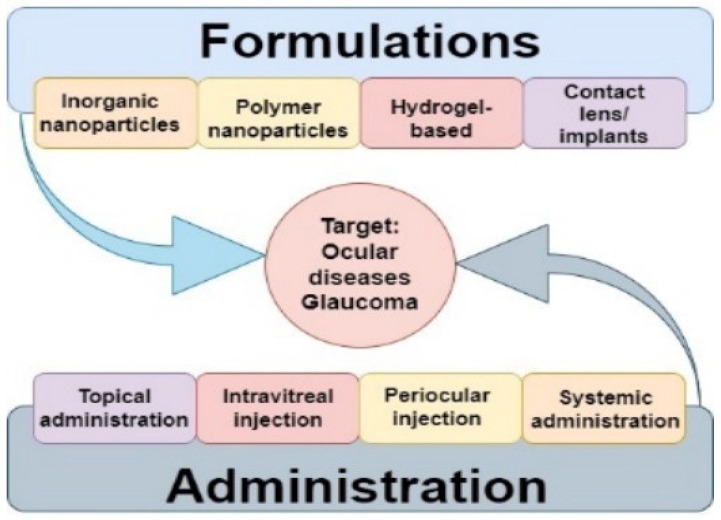
Schematic summary of drug delivery systems for glaucoma treatments.

Ordinarily, the noninvasive methodologies incorporate topical eye drops, ointments, and oral drugs, while surgical nanotechnology has enabled glaucoma drainage through inserts, laser treatment procedures, and trabeculectomy. A schematic summary of current drug delivery methods and formulations is presented in Figure 4. However, these methodologies have drawbacks [30].

Despite their efficiency in lowering the IOP, these therapeutic approaches have some important adverse effects. For example, drawbacks associated with oral medication in the treatment of glaucoma include conjunctival hyperemia in certain formulations. One of the most common treatments among oral medications is topical eye drops. However, this treatment is subject to low patient compliance and low bioavailability [31]. Alternatively, laser and surgical methodologies such as inserts and trabeculectomy have been found to be effective. However, they also carry adverse limitations, such as swelling, soreness, dryness of the cornea, and post-surgical complications [32].

In light of this, searching for novel targets to treat IOP and play a neuroprotective role could be taken as an advancement in the treatment of glaucoma by decreasing the side effects of the currently available drugs. To counteract these hindrances, targeted drug delivery systems that simplify ophthalmological treatments of glaucoma for an extended duration after the administration in the anterior and posterior parts of the eye have been designed to be sustainable [33]. To elevate bioavailability or alleviate chronic visual impairments, advanced drug delivery mechanisms should be used. Liposomes, microneedles, niosomes, dendrimers, ocular inserts, nanoparticles, and in situ hydrogels assume a significant role in upgrading the remedial adequacy of the anti-glaucomic drugs [34].

## 5. Constraints of Current Glaucoma Drug Delivery Treatment

The drug delivery of anti-glaucoma medications and therapies is challenging because of the presence of ocular barriers, which result in low bioavailability of the active ingredient within the drug [9]. Essential challenges in the administration of ocular medications through conventional strategies incorporate the lack of patient training for the technique in terms of medication, consistency, adherence, and diligence. Each treatment approach has its limitations, and there are severe side effects of some applications.

### 5.1. Eye Drops and Eye Ointments

Eye drops are a fundamental type of topical administration due to their ease of application, favorable cost, and good patient consistency [35]. The typical retention time of eye drops administered topically in the pre-corneal tear film is about 1 min. This retention time is the only time presented for drug permeation through the cornea to reach the aqueous humor. Due to obstruction of the cornea and pre-corneal components, under 5% of completely directed medications permeate to the aqueous humor. The corneal epithelium, which contains different desmosomes and tight intersections, forestalls the permeation of particles bigger than 500 Da, barring them from infiltrating the cornea [36].

Subsequently, 80% of the conveyed drug is unable to enter the cornea, and might be absorbed into the veins of the conjunctiva. Just under 10% of the given medication is uptaken into the ocular system, and roughly 1–7% of that arrives at the target site—particularly in the aqueous humor [37]. However, the most widely prescribed anti-glaucoma medications—the PG analogs—do not suffer from these same limitations, and can be effectively administered once daily, with good effects in many patients. Some drugs can infiltrate the cornea, and are immediately separated through the trabecular meshwork. In the trabecular meshwork, most ocular drugs possess a half-life slightly less than 2 h, which is a hindrance for drug molecules in reaching the targeted tissue. Regularly utilized eye drops and ointments have little corneal penetrability, and are thus restricted to treatment in the external fragment of the eye. Subsequently, to achieve the ideal dosage using this approach, eye drops should be controlled with high-recurrence dosing regimens. Consequently, using eye ointments to attain the required dosage to the posterior chambers may cause harm to the ocular cells. Patient movement while administering the eye drops and regular medication bring about poor patient compliance, and making sure to take a daily dose of the optical drug might be a challenge for patients as well [38].

### 5.2. Trabeculectomy

Trabeculectomy is frequently associated with visual hypotony, which is a post-surgical complication due to an overabundance in the filtration of fluid humor after a medical procedure. Nonetheless, surgical procedures are restricted to treatments—for example, corneal transplant, glaucoma treatment, or removal of the vitreous humor. Surgical drainage inserts are utilized in glaucoma treatment when IOP-decreasing medications cease to work. A common postoperative complication after implantation of these devices is the development of fibrosis around the implants [39,40].

### 5.3. Laser Treatment

For patients who are unable to endure the administration of other forms of medication, or for whom the therapeutic drug alone has not been satisfactory, laser treatment is an alternative. Laser treatment is becoming more common—particularly in Europe—for the treatment of glaucoma.

Subliminal trans-scleral cyclophotocoagulation (SL-TSCPC) is one of the alternative therapies to decrease IOP safely and efficiently. However, there are few studies regarding SL-TSCPC using a Supra 810 laser machine, and limited data regarding its effectiveness in moderate-severity glaucoma that still has good preservation of vision. SL-TSCPC is a safe and alternative method of lowering IOP in moderate-to-advanced glaucoma over 6 months of follow-up. As it has a good safety profile and repeatability, it is a good treatment option for patients with uncontrolled glaucoma. The parallel effect while using laser treatment is usually temporary, and may cause swelling, soreness, dryness of the cornea, and/or risk of corneal scratching by the laser [41].

### 5.4. Oral Medication

As it is rare for oral medications to be administered to glaucoma patients, when applied, the potential side effects include metallic taste, depletion of potassium, and development of kidney stones [42].

## 6. Current Pharmaceutical Interventions for the Treatment of Glaucoma

A number of medications are currently in use for the treatment of glaucoma. Typically, medications are intended to decrease elevated IOP and prevent loss of optic nerve fibers. Generally, drugs used in the treatment of glaucoma are classified by their active ingredient. These include prostaglandin (PG) analogs, β-blockers, α-adrenergic agonists, carbonic anhydrase inhibitors, and rho-kinase inhibitors. Combination drugs are also available for patients who require more than one type of medication. With various technological advancements, some drugs of various classes—including carbonic anhydrase inhibitors, PG analogs, β-blockers, miotics, α-adrenergic agonists, and hyperosmotics—have been developed. These drugs are responsible for treating glaucomatous complexes either by increasing aqueous humor drainage from the eye or by reducing aqueous humor production [43].

### 6.1. Beta-Adrenergic Blockers

Timolol maleate (TM) is the primary line of medication in the treatment of glaucoma [44], belonging to the class of β-adrenergic blockers. Even after the appearance of the most recent medications, such as PG analogs and α-2 agonists, TM remains the best option because of its cost-effectiveness. Long-lasting treatment with skin drops is typically needed in the treatment of glaucoma.

Consequently, a decrease in dosing recurrence can improve tolerance consistency and treatment. Having low blood pressure, fatigue, and a low pulse rate are the side effects of the medication. β-blockers can also be a reason for shortness of breath in people who have a history of asthma or other respiratory disorders, and can alter cardiac activity by decreasing the amount of blood the heart pumps out, which may reduce the pulse rate and/or slow down the heart’s response rate during rare side effects of exercise, including reduced libido and depression [45,46].

### 6.2. PG Analogs

PG analogs are another course of visual hypotensive medications produced for the treatment of POAG. Latanoprost and unoprostone are medications that lower the IOP specifically by increasing the uveoscleral drainage. The standard dosage of PG that reduces IOP by 30% in glaucomic patients is 50 μg/mL, applied topically once per day. Additional therapeutic impact is achieved when PGs are used with other glaucoma treatments. Potential side effects include eye color change, eyelash growth, droopy eyelids, darkening of eyelid skin, sunken eyes, stinging, eye redness, and itching [47].

### 6.3. Alpha-Adrenergic Agonists

Alpha-adrenergic agonists are usually applied after ocular laser therapy to decrease the aqueous humor secretion and to control the adverse increases in IOP and episcleral venous pressure. Unfortunately, they can induce ocular irritation and dry eyes, along with systemic side effects involving the central nervous system, and are therefore usually not recommended for long-term therapy [43].

### 6.4. Carbonic Anhydrase Inhibitors

Carbonic anhydrase inhibitors are sulfonamide derivatives that decrease ocular pressure by lessening the production of intraocular fluid, thus reducing the formation of aqueous humor and inhibiting the activity of carbonic anhydrase in the ciliary process of the eye, consequently decreasing the IOP. They are available and administered in the form of eye drops and as pills. Systemic use of carbonic anhydrase inhibitors reduces the IOP by approximately 40%. Utilizing these medications causes a quick impact on the therapeutic treatment of acute angle-closure glaucoma. Loss of strength of the hands and feet, along with tingling, upset stomach, mental fuzziness, memory problems, depression, kidney stones, and frequent urination, are among the side effects of the pill form of these medications. Side effects of the eye drops include stinging, burning, and other forms of eye discomfort [48].

### 6.5. Miotic Agents

Treatments using miotic agents primarily decrease pressure in the eye by increasing the drainage of intraocular liquid through the trabecular meshwork. Miotics work by constriction of the ciliary muscle, fixing the trabecular meshwork and permitting an expanded surge of fluid through the customary pathways. Miosis results from the activity of these medications on the pupillary sphincter. Patients who use these medications complain of dim vision, especially at night or in darkened areas such as movie theaters. This is due to constriction of the pupil. Miotics increase the drainage of intraocular fluid by making the pupil smaller, thereby increasing the flow of intraocular fluid from the eye [49].

### 6.6. Hyperosmotic Agents

Hyperosmotic agents essentially lower the IOP by causing an osmotic inclination between ocular fluid and blood. Systemic adverse effects include nausea, vomiting, headache, increased thirst, chills, fever, confusion or disorientation, electrolyte imbalances, and urinary retention [50].

## 7. Natural Polymer-Based Hydrogels as Drug Delivery Vehicles for Glaucoma Therapy

Biopolymers have been extensively investigated in a number of medical fields, including tissue engineering and drug delivery. This is largely due to the fact that they are biodegradable within the body, and do not induce an inflammatory reaction [51]. A summary of some polymers used in anti-glaucoma drug delivery systems can be seen in Section 7.9. Polynucleotides such as nucleic acids (DNA and RNA), proteins such as polypeptides, and polyesters derived from both plants and animals are also used [52].

When compared to synthetic polymers, naturally occurring biopolymers and their derivatives have acquired preference, and have a comprehensive range of applications in pharmaceutical as well as biomedical research. Natural biopolymers are preferred for medical applications due to their biodegradability, biostability, biocompatibility, and non-toxicity [53]. Additionally, natural polymers have the advantage of being readily available, economically friendly, and ecofriendly. Hydrogels designed from natural polymers exhibit high potential as drug delivery systems for biomaterials to treat ocular impairments [54,55].

The current market is brimming with numerous formulations and applications of biopolymers that are intended to treat glaucoma. Every one of the present modes and applications of drug delivery utilizes a particular biopolymer. The advantages and disadvantages of various natural biopolymers are tabulated in Section 7.9.

### 7.1. Silk Fibroin

*Bombyx mori* silk is a natural biopolymer obtained from arthropods and lepidopteran insects, particularly silkworms and some spider groups, that produce silk fibers at large. Due to their remarkable mechanophysical and biological properties, silk fibers have attracted the interest of researchers [56], for biomedical and pharmaceutical applications. Silk fibroin is an essential biopolymer used in biomedical applications due to its adaptable properties, with a natural physiology that makes it preferable in the study of tissue reconstruction in age-related ocular disease [57]. Silk fibroin is a fibrous protein that exhibits favorable biocompatibility, bioresorbability, low immunogenicity, and hydrophilicity, promoting its increasing consideration in hydrogel design. It is also rich in β-sheet structures, owing to hydrophobic domains that influence its biodegradability rate [58], as well as its cytological compatibility [59]. Silk fibroin proteins have been used for ocular therapies such as wound healing [60], ocular drug delivery [61], and ocular prostheses [62].

### 7.2. Chitosan

Linear-structured chitosan is a natural biopolymer composed of an acetylated unit of N-acetyl-D-glucosamine and β-(1→4)-linked D-glucosamine, a deacetylated unit. It is prepared by treating chitin shrimp shells and various crustacean shells with sodium hydroxide [63].

Due to poly-oxy salt formation, chitosan exhibits basic properties different from those of other polysaccharides [64]. As with other polymers, chitosan can also form hydrogels, films, and particles that can be used for biomedical applications in terms of drug delivery units, tissue engineering, cell culturing, and platforms for cancer diagnosis. Its low toxicity, high biocompatibility, and easy degradability in a natural environment which makes it suitable as natural extracellular matrices [65].

According to surveys carried out by several researchers, the major constraint when working with injectable hydrogel preparations is regulating the time of gelation [66]. However, a chitosan-based formulation of injectable hydrogel was developed that regulates the time of gelation [67].

To be used as a biomaterial, chitosan has important properties that mimic the extracellular matrices of cells, tissues, and organs. Chitosan is prepared and used either in a dried form or in the form of gels, depending on the temperature used and the amount of water present in the structure, which impart properties of flexibility [68].

According to the study conducted by Franca, J.R. et al. [69], chitosan can be widely applied in the treatment of glaucoma-induced intraocular pressure, acting as a basis for controlled drug delivery in the eye. This is because chitosan is polycationic by its very nature, allowing interaction with the polyanionic surface through hydrogen bonding of the ocular mucosa. Chitosan has several biological properties that make it an attractive material for use in ocular formulations [70]. Chitosan has inherent antimicrobial and mucoadhesive properties [71], as well as low toxicity, biodegradability, biocompatibility, and hemocompatibility [72]. Chitosan can disrupt epithelial tight junctions, thus acting as a permeability enhancer [71].

### 7.3. Alginic Acid

Brown algae are the main source of the naturally derived polysaccharide alginic acid (Alg), with the molecular formula (C_6_H_11_NO_6_)_n_. The molecular structure and composition of alginic acid consist of L-guluronic acid and D-mannuronic acid structures connected with alpha-1,4 bonds [73]. As a result of the carboxyl group attached to the C-5 carbon as a chain, it exhibits an acidic nature, and with properties such high hydrophilicity, the capacity for gelation, and pH-dependent viscoelasticity. Furthermore, biocompatibility and biodegradability are some of the physiological properties that make it suitable for use as films and gels developed for medical and food applications [74]. Alginic acid has a biodegradable and biocompatible nature that is favorable for researchers; therefore, its use has been encouraged in ocular treatments [75]. Ocular delivery therapeutics are a current trend in ophthalmology, and alginates have been employed to play an imperative role because of their biocompatibility and immunogenicity [76].

### 7.4. Pullulan

Pullulan is a non-ionic polysaccharide extracted from the fermentation of black yeast (*Aureobasidium pullulan*), and is used broadly in biomedical applications because of its less immunogenic reaction, along with its non-toxic, non-mutagenic, and non-carcinogenic nature [77]. It is utilized in the targeted delivery of drug mechanisms, tissue engineering therapy, and wound-healing activities. Pullulan responds to external stimuli so that it can be used to design hydrogels, which can be used to deliver drugs, nutrients, and (any) other molecules to a targeted area of the host [78]. The biological properties of pullulan include high water retention, biocompatibility, cytocompatibility, protective activity against microbes and biodegradation, and tissue-regenerative characteristics [79].

### 7.5. Hyaluronic Acid

A biopolymer regularly found and extracted from the human body, applications of hyaluronic acid as injectable hydrogels have been researched for ocular drug delivery systems, since they can be designed as both stimulus-responsive and static [80]. Anionic hyaluronic acid is incapable of gelation without additive molecules. Hence, hydrogels produced using hyaluronic acid depend on chemical modifications. Egbu et al. formulated two hyaluronic acid gel systems embedded with infliximab for the treatment of blinding infections influencing the elderly population [81]. Hyaluronic acid has been applied in ocular therapeutics because of its favorable biological characteristics, such as biocompatibility, biodegradability, and non-immunogenicity [82]. Due to hyaluronic acid’s biological safety, it has various ophthalmology-related applications, such as treatment for dry eyes, intravitreal drug delivery, and use in contact lenses [82].

The main objective in the development of ophthalmic drug treatment is to extend the therapeutic extent of medications, particularly proteins and antibodies [83].

### 7.6. Dextran

Dextran methacrylate and cyclodextrin–dextran are a few examples of dextran hydrogels used in ocular drug delivery [84]. Properties such as stiffness, mechanical strength, and solidness can be adjusted by regulating the monomer in the gel, subsequently improving their significance in drug delivery. Yao et al. [85], designed a drug delivery system for effective in vivo drug release of bevacizumab from a hyaluronic acid/dextran-situated in situ hydrogel for 6 months after intravitreal infusion in hare eyes. The in vivo drug release efficiency results indicated that bevacizumab was delivered at a therapeutically relevant concentration by means of a controlled release mechanism within the vitreous humor [86]. Dextran has been found to exhibit great biocompatibility and low cytotoxicity. Additionally, it has hydrophilic domains, which promote its biodegradability in water and other organic solvents. This biological feature enables its applicability in blended forms with bioactive agents of hydrophobic polymers [87].

### 7.7. Methylcellulose

Derived from cellulose, hydroxypropyl methylcellulose (HMPC) is widely used in the pharmaceutical industry because of its solvency in water, rheological properties, and transparency [88]. A group of researchers designed a trans-scleral antisense oligonucleotide-loaded gel for the delivery of drug-loaded macromolecules using methylcellulose and ι-carrageenan dispersions [89]. Periocular injection of the gel resulted in impressive choroid and sclera bioavailability in comparison to the injection of an oligonucleotide solution alone. Methylcellulose has been incorporated into ocular inserts of three types: soluble, insoluble, and bio-erodible, [90]. Methylcellulose has low reactivity with cells. Additionally, interest has been shown in mixing it with biologically active materials such as cytokines and/or the extracellular matrix to control the organization or functions of the cells [91].

### 7.8. Gelatin

Gelatin is a collagen-derived biopolymer normally found in scleral and corneal stroma, and its structural networks make it an attractive natural complex for research applications. El-Feky et al. [92] developed an oxidized sucrose-crosslinked gelatin–chitosan hydrogel with the end goal of TM drug conveyance for the treatment and control of ocular hypertension [93]. In vivo and in vitro discoveries indicated that the formulated system maintained favorable release efficacy of the active ingredient, in contrast to the regular eye drops [94]. Gelatin has favorable biological characteristics such as low antigenicity, biocompatibility, and biodegradability, and promotes cell proliferation; therefore, it is widely researched in ophthalmologic therapeutics [95].

### 7.9. Collagen

Collagen is biocompatible, biodegradable, and non-toxic for living organisms [96]. Type 1 collagen is an essential biopolymer that has been utilized in hydrogels for tissue engineering applications [97]. Wong et al. [41] designed an injectable composite comprising collagen and alginate for retinal treatment through a drug delivery system loaded with an ocular drug. A summary of polymers used in anti-glaucoma drug delivery systems discussed in Table 1. Table 2 presents some natural biopolymers used in ophthalmic injectable hydrogels. Intravitreally infused gels exhibited adequacy in rodents with deteriorating retinae and photoreceptor apoptosis. Twofold portion infusions into a similar eye yielded greater results without sacrificing the gel’s feasibility [98].

## 8. Design of Hybrid Hydrogels for Injectable Drug Delivery in the Treatment of Glaucoma

The development of hybrid hydrogels should be conducted in a sterile environment that is free from the creation of an overabundance of gas, protons, heat, or substances dangerous to living beings. The gel should be shaped under physiological states of temperature and ionic strength, light, and chemical gelation, in a controllable manner [105,106].

A hydrogel ought to have the capacity to conform to various applications, and should have the option to be administered via a slight needle (i.e., 30-gauge or thinner) in the confined space of the eye. Other fundamental variables to be considered are the mode of crosslinking, the solvents used in biopolymer dissolution, the solvent and chemical molecular weight and concentration, and the crosslinking time period [107,108].

### 8.1. Physicochemical, Pharmacokinetic, and Pharmacodynamic Properties of Ophthalmic Hydrogels

To design this intravitreal injectable hydrogel system, there should be strict parameters adhered to, such as viscoelasticity, viscosity, drug release efficiency, sustainability, etc. [109,110].

#### 8.1.1. Drug Release Efficiency

Hydrogels developed for targeted drug delivery should have the ability to encapsulate a highly concentrated drug with a sustained release profile from the crosslinked hydrogel, so that an initial burst release is inevitable [111]. A high local concentration of the active pharmaceutical ingredient is retained over a significant stretch of time by means of a suitable release mechanism controlled by swelling, diffusion, or chemical/environmental stimuli [112].

Covalently crosslinked hydrogels have been utilized in the development of ocular drug delivery systems. These hydrogels remain in situ if applied topically to the lacrimal canal; however, if administered intravitreously, they tend to display swelling mechanisms. The drug release efficiency of embedded drugs can be modulated to some degree by variance in biopolymer concentration and molecular weight, adjustment of crosslinking density, and alteration of the degradation rate. Modulating these parameters may also alter other parameters of the drug–complex system, such as biocompatibility, mechanical properties, and the stability of the active ingredient [113].

#### 8.1.2. Biocompatibility

Biocompatibility testing provides initial screening of whether or not the components of the desired biomaterial may cause adverse effects when interacting with the human body. These biocompatibility tests may include sensitization assays, carcinogenesis, hemocompatibility, genotoxicity, and systemic toxicity [111]. To design an injectable hydrogel, biocompatibility with cells, bodily fluids, and tissues should be considered in light of the fact that the hydrogel should maintain cell differentiation without causing cytotoxicity or adverse inflammatory responses in the host organism. Subunits of most biopolymers derived from natural sources are similar to organic extracellular matrix (ECM), making them more biocompatible than manmade polymers [114].

#### 8.1.3. Biodegradability

Naturally derived biopolymers with the ability to degrade to naturally occurring byproducts without causing adverse inflammatory and immune responses are preferred for use [115]. The hydrogel ought to be biodegraded, bio-eroded [116], and expelled from the host at a rate that is relative to the pace of the tissue/organ development, so as to make space for cell multiplication [117].

#### 8.1.4. Porosity

Exceptionally interconnected and profoundly coordinated permeable networks that exhibit micro- or nanoscale pores are preferred in hydrogel design and development [118]. The partial permeability facilitates active ingredient release of the therapeutic drug from the hydrogel, and supports cell nutrient movement for cell growth [119].

#### 8.1.5. Viscosity

Low hydrogel viscosity is a crucial factor when designing these biomaterials, because the hydrogel should permit a homogeneous distribution of the active ingredient prior to complex gelation [120].

#### 8.1.6. Mechanical Strength

A hydrogel ought to offer mechanical support and guide cell differentiation [121,122]. Mechanical strength is significant because the biomaterial must be able to withstand its biochemical structure and shape to overcome unavoidable natural forces that come with the eye’s physiology [123].

#### 8.1.7. Swelling Properties

A favorable property of hydrogels is their capacity to grow in contact with a thermodynamically viable solvent [124,125]. At the point when a hydrogel in its underlying state is in contact with dissolvable particles, the latter assaults the hydrogel’s surface and infiltrates into the polymeric organization [126]. Solvent molecules penetrate into the polymeric network due to charge repulsion between polymer chains, causing an increase in the polymer volume due to liquid uptake [127].

#### 8.1.8. Rheology

Rheology instruments can be utilized to gauge antecedent arrangement attributes, e.g., yield pressure, which have direct importance for clinicians’ utilization of the materials [110,128]. Practically speaking, the three most applicable rheological boundaries are the simplicity of infusion (shear reaction), time for position (recuperation time), and maintenance of the hydrogel’s forerunner arrangement at the deformity site (yield pressure) [129].

#### 8.1.9. Opacity and Transparency

Hydrogels that bio-mimic the high opacity and transparency of the natural ocular humor remain materials of preference [130,131].

## 9. Intravitreal Administration of Injectable Drug-Loaded Hydrogels to The Eye

Ophthalmic applications of intravitreal injectable drug-loaded hydrogels for glaucomic treatment to the posterior section of the eye have been shown to overcome ocular barriers and effectively treat glaucoma [132,133]. This novel drug delivery system has properties of good adherence and viscosity, and achieves the objective of retention and therapeutic treatment of ocular diseases in the most remote areas of the eye.

To counteract ocular barriers and defense mechanisms, scientists have developed intravitreal injectable hydrogel-based drug delivery mechanisms that enable retention on the surface of the eye and in the posterior segments of the eye for an extended duration after their administration, where conventional therapies have not successfully and sustainably achieved the same targets [134]. Administration of intravitreal injectable hydrogels results in an acceptable retinal bioavailability, since the drug is directly injected into the targeted area [135]. An advantage of administering intravitreal injectable hydrogels is that they are biodegradable, so they are exceptionally alluring as intravitreal delivery systems because they are degraded by the body over a favorable timeframe that relies upon the particular polymeric framework utilized [136].

The installation of this type of delivery system is just one methodology. For example, the drug-loaded polymeric complex will be required once as an injection to the target site, whereafter the polymer will undergo optimal biodegradation, bio-erosion, and elimination from the body, without creating any possible incendiary side effects. Intravitreal injectable hydrogels alleviate the problem faced while using solid implants, because injectable hydrogels can hold water. In addition, their easy dissolution enables them to encapsulate drugs to be released in the local area. The administration of intravitreal hydrogels as drug delivery systems increases the bioavailability of the active drug at the required target site by overcoming ocular barriers [137].

Due to their biocompatibility and suboptimal associated inflammatory response, intravitreal hydrogels today have become a potential solution to current treatment complications, especially for preventable neural retinal diseases such as macular degeneration and glaucoma. There are several treatments that are used for such therapy. However, many of them exhibit various problems and limitations. In order to be able to solve these drawbacks, the use of injectable hydrogels as drug delivery materials can improve the success of the therapy [138].

Non-expulsion of a depleted non-biodegradable embed may aggravate the visual tissue. Thus, it is better if it is eliminated after the medication is depleted. There is no requirement for expulsion after the medical procedure. This implies that there will be a lesser danger of difficulties related to the significant visual medical procedure. Trends in systems have seen in situ gel frameworks become an exploration hotspot—particularly for improvements in responsive hydrogels, such as ion-sensitive hydrogels, thermo-sensitive hydrogels, and pH-sensitive hydrogel [139].

### Alternative Injection Locations

Intravitreal injection is one of the current methods of pharmaceutical delivery mechanisms performed as often as monthly, which can result in resistance [116]. Currently, there are alternative intraocular injections with respect to the location and drug being delivered that might be more useful [140]. These alternatives include sub-conjunctival injection and parabulbar injection, which take place underneath the conjunctiva for trans-scleral delivery [141]. Practically, a sustained in vivo drug release for up to 4 weeks was shown following sub-conjunctival injection of dorzolamide-loaded polymer disks [142]. Dorzolamide is a carbonic anhydrase inhibitor, and it works by decreasing the pressure in the eye [143].

Another alternative is sub-retinal injection, which is delivered beneath the retina to the sub-retinal fluid. Sub-retinal injection has more direct effects on the targeted cells in the sub-retinal space, providing a new therapeutic method for vitreoretinal diseases—especially when gene therapy and/or cell therapy is involved [144]. Via this effective technique, clinicians can administer drugs such as anti-VEGF [145] and steroids [146], among others, directly into the back of the eye to increase the drug concentration in the vitreous humor and the retina.

## 10. Pharmacokinetics of Intravitreal Hydrogel Drug Release

With this approach, the drug-loaded polymer complex is administered intravitreously at the targeted posterior segment of the eye via a minimally invasive procedure. The injectable systems utilize an anti-glaucoma drug-loaded polymer drug delivery system [36]. The polymer derived from ethylene-vinyl acetic acid is known by its non-degradable nature, which prompts a resistant reaction because of the extended presence of an unfamiliar body. The pace of drug release of medications from these frameworks is variable [147].

The water solvency of the medication influences its adequacy, on the grounds that hydrophilic medications cooperate ineffectively with biodegradable polymers due to their hydrophobic nature. The water solubility of some drugs has a direct influence on the drug release efficacy due to their hydrophilic nature [148].

Once the anti-glaucoma drug-loaded hydrogel has been injected into the target, the drug molecules are distributed by Fickian diffusion from the vitreous humor to the surrounding ocular tissues within reach and the target retinal sites. The vitreous body is not a hindering factor of drug diffusion of some soluble proteins; however, it limits the mobility of various drug delivery systems, such as drug-loaded nanoparticles. The vitreous humor facilitates an increased rate of low-molecular-weight drug diffusion, since the mesh size in the vitreous network has been estimated to be ~500 nm. Drugs administered intravitreally normally have a diameter of 10 nm and below, e.g., ranibizumab (4.1 nm) and bevacizumab (6.5 nm). Distribution of therapeutic drugs and the biodegradation of self-assembled polymers and nanoparticulate transporters have previously been confirmed [149].

Intravitreal injection of the biopolymer–drug complex into the sub-conjunctival space and posterior target site can lead to prolonged delivery over weeks or months, compared with simple topical application, which would last at most of a few hours or days [54]. Biodegradable and non-biodegradable polymers have been investigated for their application as injectable hydrogels for ocular treatment and therapy. Poly(ethylene-co-vinyl acetate)—a non-degradable polymer—exhibits extended drug delivery efficiency for a wide range of active drug ingredients, but unfortunately exhibits poor biodegradability, and the continued retention of implanted foreign body material causes immune responses. On the other hand, biodegradable polymers such as silk fibroin, chitosan, and alginate may be the best alternatives for intravitreal injection, as they are more suitably biodegradable [150].

## 11. Discussion

In this review, various therapeutic anti-glaucomic drug delivery systems were investigated, and the most promising ones were highlighted. This survey elaborates the importance of using natural biopolymer-based injectable hydrogels rather than other therapeutic drug delivery systems utilizing synthetic materials, because of the former’s biodegradability, biocompatibility, and non-immunogenic properties.

Numerous studies have proven that natural biopolymers rarely cause adverse inflammatory and immune responses when in contact with ocular tissue, as opposed to synthetic polymers. The therapeutic injectability of these drug-loaded polymer complexes has successfully overcome anatomical and physiological ocular barriers, thus increasing bioavailability and therapeutic efficacy. Unlike other therapeutic drug delivery systems that have failed to permeate through the ocular barriers, injectable hydrogels have the potential for prolonged sustainability in the delivery of ocular drugs. The potential improvement in patient compliance and persistence for optimal outcomes with the help of these systems is unprecedented.

## 12. Conclusions

The development of intravitreal injectable hydrogel drug delivery systems is a promising approach for the treatment of ophthalmological diseases—particularly glaucoma. Ocular treatment remains challenging for scientists because of physiological ocular barriers to any foreign substances. Thus far, using intravitreal injectable hydrogels for the treatment of ocular disease has facilitated the delivery of drugs to the targeted area at the desired dosage, with improved properties of penetration, bioavailability, and extended retention time for the release of the drugs. All of this has been achieved by encapsulating drugs into hydrogels made from naturally obtained biopolymers. Therefore, the assessment presented in this review indicates that hydrogels made from natural biopolymers have the ability to overcome the limitations of conventional ocular treatment, and could hence become potential sources and suitable matrices with excellent biocompatibility, acting as useful vehicles for the delivery of drugs. Researchers should devote more attention to the production and application of intravitreal hydrogels made from natural polymers to deliver drugs to targeted areas for the treatment of glaucoma.

## Figures and Tables

**Table 1:** Summary of some polymers used in anti-glaucoma drug delivery systems.

Polymers	Delivery System	Drug Used	Feature	Reference
Silk fibroin	Nanoparticles	TM	TM caused a sustained and prolonged reduction in IOP without adverse effects on the physiology of the eye compared to conventional free drug use.	[99]
Hydroxyethyl chitosan	Hydrogel	Heparin	The heparin-loaded hydroxyethyl chitosan hydrogel was able to sustain and improve the reduction in the IOP after GFS for protracted periods of time. Clear inflammatory responses and results were not seen in the eye during the trial’s timeframe.	[100]
Gelatin-*g*-poly(*N*-isopropylacrylamide)	Hydrogel	Pilocarpine	Pilocarpine-loaded gelatin hydrogels were designed by grafting with carboxylic end-capped poly(N-isopropylacrylamide) for anti-glaucoma treatment by intracameral administration.	[101]
Poly (lactic-co-glycolic acid) (PLGA)	Nanoparticles	Dexamethasone and melatonin	A dual-loaded melatonin and dexamethasone poly(lactic-co-glycolic acid) nanoparticle system was designed as an anti-glaucoma treatment option. The in vitro release of the loaded drug from the nanoparticles revealed a supported delivery profile for the two medications, with no signs of burst discharge.	[102]
Gellan gum/pullulan	Nanofibers, in situ gel	Fluorescein sodium	Development of a novel fluorescein delivery system that is applied topically in dry nanofibrous form and gelates in situ immediately after administration guaranteed a solid match to the eye structure by the designed nanofibers, which were molded into conforming geometries. Prolongation of the ocular drugs’ residence time was achieved	[103]
Chtosan/hydroxyethyl cellulose	Ocular inserts	Dorzolamide	Dorzolamide-loaded ocular inserts were effective in glaucoma treatment. The ophthalmologic drug embedded in the polymeric matrix displayed a 3-h drug release efficiency, and released 75% of the loaded drug.	[104]
Alginate–chitosan	Nanoparticles/nanogels	TM	This study proposed that loading and delivering TM onto alginate–chitosan nanoparticles may be a suitable drug delivery approach for controlled delivery of TM through the cornea	[99]

**Table 2:** Natural biopolymers used in ophthalmic injectable hydrogels.

Natural Biopolymer	Gelation	Strengths	Drawbacks	Reference
Silk fibroin	Ionic crosslinking, hydrophobic interactions	Easily modified	Low mechanical strength	[53]
Chitosan	Chemical crosslinking, pH gelation	Simple to adjust	Low dissolvability at neutral pH	[54]
Alginate	Chemical gelation, ionic crosslinking	Favorable mechanical properties, rapid gelation	Poor cytoadhesion	[55]
Gelatin	Chemical crosslinking	Hydrophilic, various responses available	Susceptible to degradation, poor mechanical properties,	[94]
Pullulan	Chemical crosslinking	Easily dissolvable	Undesirable swelling properties and mechanical properties	[77]
Methylcellulose	Hydrophobic, chemical, physical	Easy modification of physiochemical properties	Uncontrollable degradation, poor cell adhesion, poor mechanical properties	[74,89]
Dextran	Chemical crosslinking, physical crosslinking	Simple crosslinking, large capacity, hydrophilic, controlled drug release	Prone to causing in vivo side effects	[71,90]
Hyaluronic acid	High temperature (specific to contact with other polymers), chemical gelation	Simple modification, natural vitreous component (ECM), bioactive	High viscosity, susceptible to degradation	[80]
Collagen	Chemical crosslinking, high temperature	Natural ECM component, favorable cell adhesion,	Susceptible to degradation, strenuous dissolution	[85]

## Data Availability

Not applicable.
